# Ultra-Performance Liquid Chromatography and Mass Spectrometry Characterization, and Antioxidant, Protective, and Anti-Inflammatory Activity, of the Polyphenolic Fraction from *Ocimum basilicum*

**DOI:** 10.3390/molecules29215043

**Published:** 2024-10-25

**Authors:** Magdalena Wójciak, Roman Paduch, Piotr Drozdowski, Magdalena Żuk, Weronika Wójciak, Katarzyna Tyszczuk-Rotko, Marcin Feldo, Ireneusz Sowa

**Affiliations:** 1 Department of Analytical Chemistry, Medical University of Lublin, 20-093 Lublin, Poland; magdalena.zu25@gmail.com (M.Ż.); weronikawojciak01@gmail.com (W.W.); i.sowa@umlub.pl (I.S.); 2 Department of Virology and Immunology, Institute of Biological Sciences, Faculty of Biology and Biotechnology, Maria Curie-Skłodowska University, 20-033 Lublin, Poland; roman.paduch@mail.umcs.pl; 3 Department of General and Pediatric Ophthalmology, Medical University of Lublin, 20-079 Lublin, Poland; 4 Department of Plastic Surgery, Specialist Medical Centre, 57-320 Polanica-Zdrój, Poland; piotr_drozdowski@wp.pl; 5 Faculty of Chemistry, Institute of Chemical Sciences, Maria Curie-Skłodowska University in Lublin, 20-031 Lublin, Poland; katarzyna.tyszczuk-rotko@mail.umcs.pl; 6 Department of Vascular Surgery, Medical University of Lublin, 20-081 Lublin, Poland; martinf@interia.pl

**Keywords:** basil, antioxidant, cytotoxicity, polyphenols, adenocarcinoma, normal colon epithelial cells

## Abstract

*Ocimum basilicum* is a valuable plant widely consumed worldwide and considered a rich source of polyphenols. This study examined the impact of the polyphenolic fraction isolated from basil (ObF) on human normal colon epithelial cells and human colorectal adenocarcinoma cells, evaluating its anti-inflammatory and protective activity against oxidative stress. The phytochemical characterization of the fraction was performed using ultra-performance liquid chromatography (UPLC) with a photodiode detector (DAD) and mass spectrometry (MS). UPLC-DAD-MS revealed that ObF predominantly contains caffeic acid derivatives, with rosmarinic acid and chicoric acid being the most abundant. The fraction demonstrated high antioxidant potential, as shown by DPPH assays, along with significant reducing power (FRAP). Furthermore, it prevented the depletion of antioxidant enzymes, including superoxide dismutase and catalase, and decreased malonylodialdehyde (MDA) in induced oxidative stress condition. Additionally, it exhibited a significant protective effect against H_2_O_2_-induced cytotoxicity in human normal colon epithelial cells. Although it had no impact on the viability of adenocarcinoma cells, it significantly reduced IL-1β levels in the neoplastic microenvironment. Our study demonstrated that basil polyphenols provide significant health benefits due to their antioxidant and protective activities.

## 1. Introduction

Polyphenols are a diverse group of plant components belonging to secondary metabolites. They can be classified into several categories, including flavonoids, phenolic acids, lignans, and stilbenes. Each group has unique structures that contribute to their biological effects. Polyphenols are widely distributed in plants and can be found in leaves, stems, roots, and fruits. Therefore, they are common constituents of the human diet, as they occur in fruits, vegetables, and plant-derived products [1,2]. The health benefits of polyphenols are extensive [3,4,5,6,7]. It is believed that regular consumption of polyphenol-rich foods is associated with better health outcomes and longevity. Most importantly, they are known for their antioxidant activity, which helps neutralize harmful free radicals, potentially reducing the risk of chronic diseases such as cardiovascular disease, cancer, and neurodegenerative conditions. Additionally, polyphenols have anti-inflammatory, anti-microbial, and even anti-diabetic effects, playing a crucial role in maintaining overall health [3,4,5,6,7].

The Lamiaceae family, which includes 150 species, is known for plants with a high polyphenol content [8]. One of them is *Ocimum basilicum* L. (Basil), which is widely cultivated around the world, especially in Mediterranean countries. It is a bushy plant with bright green, oval-shaped and aromatic leaves commonly consumed fresh or dried as it is widely used as a seasoning in various cuisines. *O. basilicum* has been recognized as a valuable plant since ancient times and is used in various traditional medical systems such as Unani and Ayurveda due to its biological properties. In traditional medicine, basil leaves are commonly used to treat ailments such as fever, cough, digestive disorders, and headaches [9,10]. In East Nusa Tenggara, and Indonesia, fresh basil leaves are frequently consumed to treat malaria [11]. In North Sumatra, the plant is also employed to treat rheumatism, high cholesterol, hypertension, headaches, and stroke [12]. Literature data confirm the biological potential of basil. It has been found that it exhibits anti-inflammatory, antibacterial, antiviral, antidiabetic, antioxidant, and wound healing properties [13,14,15,16,17,18]. There are also some reports on cytotoxic activity against human melanoma, human breast cancer, and leukemic cells [19,20,21,22,23].

It should be noted that many of the reports focus on the components of basil essential oils and their activity [9,10]; however, basil is also a rich source of polyphenols [24,25,26], which may contribute to its antioxidant and anti-inflammatory properties.

Thus, the aim of the study was to assess the antioxidant, protective, and anti-inflammatory effects of a basil polyphenolic fraction as well as its impact on human normal colon epithelial cells. Furthermore, the cytotoxic potential against human colorectal adenocarcinoma was investigated, as some studies suggest the anticancer effects of basil. The qualitative and quantitative composition of the polyphenolic fraction was established using mass spectrometry and UV-Vis detection.

## 2. Results

### 2.1. Phytochemical Assay of the Extracts

**Figure 1:**
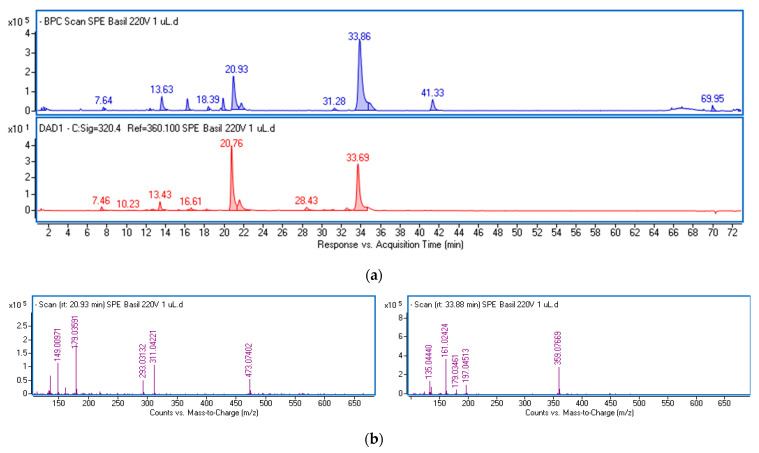
Base peak chromatogram in negative ionization mode (blue line) and chromatogram at λ = 320 nm (red line) of the phenolic fraction from ethanol–water extract of *O. basilicum* leaves (**a**) and mass spectra of the main components (**b**) identified as chicoric acid (**left** panel) and rosmarinic acid (**right** panel).

The polyphenolic fraction (ObF) was obtained through solid-phase extraction (SPE) of an ethanol–water extract from *O. basilicum* leaves. This procedure reduces the matrix of non-phenolic constituents, including less polar compounds like sterols, fatty acids, and phospholipids, as well as highly polar substances such as organic acids (Figure S1, Supplementary Materials). The phenolics in the fraction were analyzed using ultra-high-performance liquid chromatography (UPLC) and identified based on retention times, UV-Vis, and mass spectra. Chemical formulas were generated by the MassHunter software (Version 3.3.2 SP2 build 3.3.2.1037) based on mass data, and the identity was further verified using standards or reports in the literature [24,25,26]. Figure 1 displays the base peak chromatogram (BPC) and the chromatogram obtained at λ = 320 nm of the dissolved fraction, with the mass spectra extracted from the main peaks. Table S1 presents the mass data and estimated formulas (with Δ ppm) for the recorded peaks. Table 1 summarizes the spectral data and the quantification results of the main phenolic constituents in the fraction, expressed per gram of dried extract.

The predominant constituents of the fraction were various derivatives of caffeic acid, characterized by a common fragment ion (*m*/*z*-H = 179) and a characteristic absorption maximum in the 324–327 nm region (Figure S2). The main peaks were identified as chicoric acid (at 20.93 min) and rosmarinic acid (at 33.86 min). Both compounds were also found in their isomeric forms. Chicoric acid (dicaffeoyltartaric acid) exhibits a common ion with a mass of 473.07 [M-H]− and a characteristic ion pattern, including a peak at *m*/*z* 311 corresponding to the loss of a caffeoyl unit, a peak at *m*/*z* 293 due to the loss of caffeic acid, a peak at *m*/*z* 179 typical for caffeic acid, and a peak at *m*/*z* 149 representing tartaric acid (Figure 1b). The peak at *m*/*z* 135 results from the loss of CO_2_ from caffeic acid. Rosmarinic acid and its isomer display a parent ion at *m*/*z* 359.07, followed by fragment ions including *m*/*z* 161 (caffeoyl moiety), and *m*/*z* 179 and 197, which are formed by the cleavage of the ester bond between the two components of rosmarinic acid (caffeic acid and 3,4-dihydroxyphenyllactic acid), and the fragment ion at *m*/*z* 135.04, resulting from the loss of CO_2_ from caffeic acid. The total amounts of both acids and their isomers in the dried fraction were 20.4 and 29.2 mg/g, respectively.

Furthermore, free caffeic acid, caffeoylmalic acid, caffeic acid hexoside, caftaric acid (caffeoyl tartaric acid), and caffeoyl feruloyl tartaric acid with a common ion at *m*/*z* 179, were found in the extracts, with a total amount of 16.1 mg/g.

No components from the flavonoid class, including quercetin and kaempferol derivatives were detected. The detailed quantification of phenolics in the investigated fraction is shown in Table 1.

### 2.2. Impact of O. basilicum Polyphenolic Fraction on Human Normal Colon Epithelial Cells

#### Cytotoxicity and Flow Cytometry Assay

To determine the maximum concentration of the fraction that has no negative impact on the cells, several factors were considered, including cytotoxicity based on neutral red (NR) and MTT assays, cell morphology after May–Grünwald–Giemsa (MGG) staining, and the impact on the cell cycle and apoptosis rate (flow cytometry).

**Figure 2:**
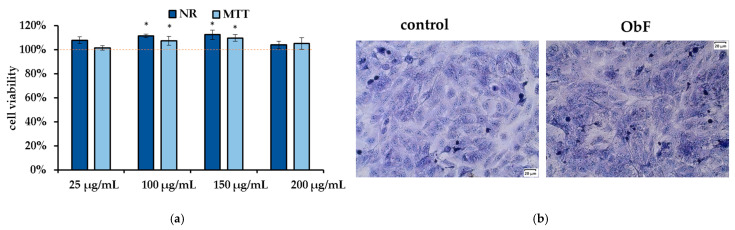
(**a**) Cell viability assessed by MTT and NR assays in human normal colon epithelial cells (841 CoTr) exposed to different concentrations of the polyphenolic fraction from *O. basilicum*. The control (0.5% DMSO in medium) is represented by the dashed red line. * indicates a statistically significant difference (*p* < 0.05). (**b**) Microscopic images of the control and the cells after 24 h treatment with the polyphenolic fraction from *O. basilicum* at a concentration of 200 µg/mL, following May–Grünwald–Giemsa (MGG) staining. Magnification 100×. Bar = 20 µm.

The neutral red (NR) assay, which measures the accumulation of neutral red dye in lysosomes by living cells, and the MTT assay, which reflects the metabolic activity of cellular oxidoreductase in viable cells, are considered complementary tests. As presented in Figure 2a, 24 h of treatment with the polyphenolic fraction in all tested concentrations did not affect cell viability or metabolism, and the number of viable cells remained at the control level. Microscopic observation of the cells also showed no negative effects of the fraction, with cells maintaining stable contact and no signs of detachment (Figure 2b).

**Figure 3:**
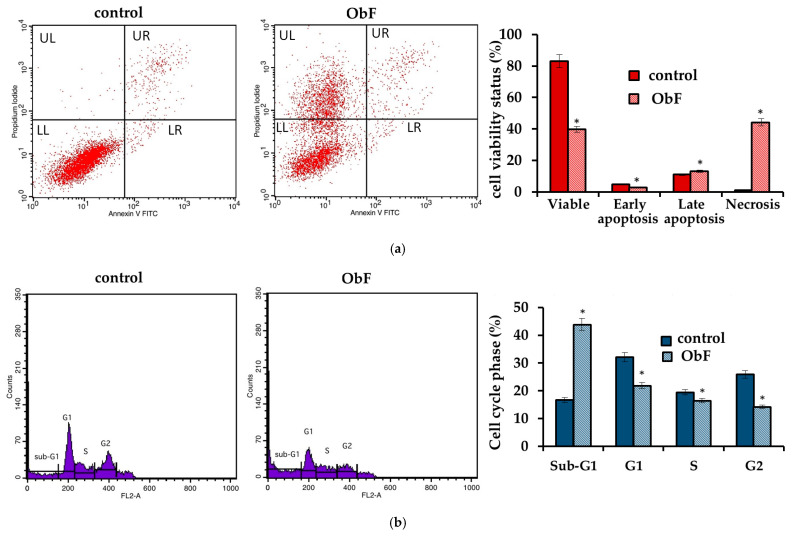
(**a**) Flow cytometry results for control and polyphenolic fraction from *O. basilicum* (ObF) (200 µg/mL) after 24 h treatment of human normal colon epithelial cells (**b**) Distribution of cells in the particular cell cycle phases. * indicates a statistically significant difference at *p* < 0.05 using one-way ANOVA followed by Dunnett’s post hoc test.

However, the flow cytometry assay, which can provide detailed information about the status of individual cells and detect early apoptosis, showed that at a concentration of 200 µg/mL, the polyphenolic fraction has a negative effect on 841 CoTr cells (Figure 3). The quantitative analysis of the cells after 24 h of treatment at this concentration showed a significant increase in the number of necrotic cells, accompanied by a decrease in viable cells compared to the control. The plot of annexin V versus propidium iodide (PI) is shown in Figure 3a. The individual regions of the plot represent the following: UL—necrotic cells (annexin V—and PI+); UR—late-stage apoptosis (annexin V+ and PI+); LR—early-stage apoptosis (annexin V+ and PI−); and LL—living cells (annexin V− and PI−).

Furthermore, cell cycle analysis showed that the fraction at 200 µg/mL caused significant changes in the cell cycle compared to the control (Figure 3b). ObF significantly increased, by almost 3-fold, the population of normal human colonic epithelial cells in the sub-G1 phase of the cell cycle and significantly reduced the population of normal cells in the G1 phase compared to the control. This suggests cycle inhibition at the border of cells entering the G1 phase. Moreover, the number of cells in the S phase was reduced after exposure to ObF. The fraction also significantly reduced the number of cells in the G2 phase, suggesting a second point of inhibition at the S/G2 interface.

No differences were observed in the flow cytometry assay between the polyphenolic fraction at lower concentrations and the control, indicating that up to 150 µg/mL of the polyphenolic fraction had no negative effect on normal human colon epithelial cells.

### 2.3. Impact of O. basilicum Polyphenolic Fraction on Human Colorectal Adenocarcinoma (HT29)

**Figure 4:**
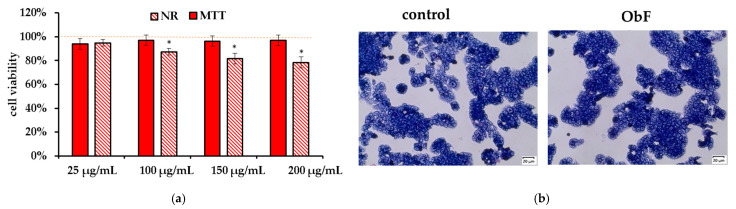
(**a**) Cell viability assessed by MTT and NR assays in human colorectal adenocarcinoma cells (HT29) exposed to different concentrations of the polyphenolic fraction from *O. basilicum*. The control (0.5% DMSO in medium) is represented by the dashed red line. * indicates a statistically significant difference (*p* < 0.05). (**b**) Microscopic images of the control and the cells after 24 h treatment with the polyphenolic fraction from *O. basilicum* (ObF) at a concentration of 200 µg/mL, following May–Grünwald–Giemsa (MGG) staining. Magnification 100×. Bar = 20 µm.

The NR and MTT assay showed that the *O. basilicum* polyphenolic fraction had only a minor effect on the viability of the HT29 cells. It also did not influence cell morphology. The cells were in stable assemblies without a tendency to loosen (Figure 4).

**Figure 5:**
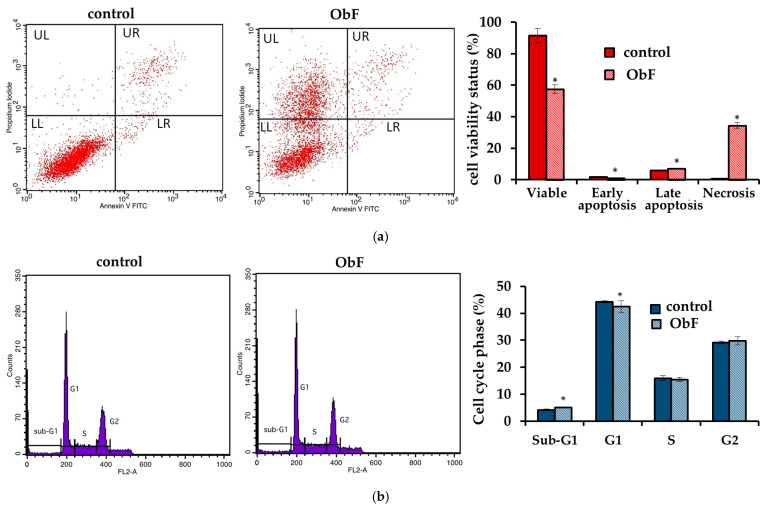
(**a**) Flow cytometry results for control and polyphenolic fraction from *O. basilicum* (ObF) (200 µg/mL) after 24 h treatment of human colorectal adenocarcinoma cells (**b**) Distribution of cells in the particular cell cycle phases * indicates a statistically significant difference at *p* < 0.05 using one-way ANOVA followed by Dunnett’s post hoc test.

However, similarly to normal cells, a disturbance of the cell cycle and a decrease in number of viable cells were observed. After a 24 h incubation with ObF at concentration of 200 µg/mL, the population of cancer cells in the sub-G1 phase significantly increased, which may suggest an increase in the number of cells in the initial phase of apoptosis and cycle limitation at the border of the G1 phase. Therefore, the number of cells in the G1 phase was significantly reduced compared to the control. The number of cells in the S and G2 phase did not change quantitatively after exposition to ObF compared to the control (Figure 5).

### 2.4. Anti-Inflammatory Effects

Cytokine concentration (pg/mL) was assessed after 24 h of incubation of both human colon tumor cells (HT29) and normal human colon epithelial cells (841 CoTr) with 150 µg/mL of the polyphenolic fraction (ObF). The investigation showed that ObF significantly reduced the level of IL-1β released by colon cancer cells (HT29 cell line), and increased IL-10, while it had no effect on the release of IL-6. Additionally, ObF did not affect cytokine levels in normal human colon epithelial cells (Table 2).

### 2.5. Free Radical Scavenging Activity and Reducing Antioxidant Power

Free radical scavenging activity was assessed based on the sample’s ability to scavenge the DPPH (1,1-diphenyl-2-picrylhydrazyl) radical. Reducing antioxidant power was evaluated based on the sample’s ability to reduce the ferric ion (Fe^3+^) complex to the ferrous (Fe^2+^) complex (FRAP). The results, expressed as Trolox and ascorbic acid equivalents, respectively, demonstrate significant antioxidant and reducing potential of the *O. basilicum* polyphenolic rich fraction (Table 3).

#### Protective Effects and Impact on the Activity of Antioxidant Enzymes

**Figure 6:**
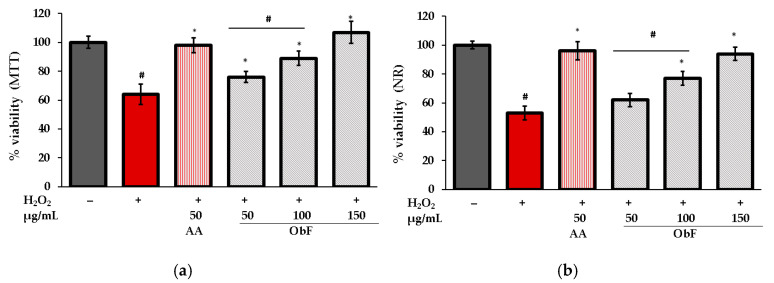
Cell viability assessed by (**a**) MTT and (**b**) NR assays in human normal colon epithelial cells (841 CoTr) pretreated with different concentrations of the polyphenolic fraction from *O. basilicum* (ObF) before exposure to H_2_O_2_. The control was 0.5% DMSO in medium. AA—ascorbic acid. * indicates statistically significant difference vs. H_2_O_2_-stimulated cells; # indicates statistically significant difference vs. untreated control (*p* < 0.05).

The protective activity of the polyphenolic fraction from *O. basilicum* against oxidative stress was assessed by estimating cell viability in cells stimulated by H_2_O_2_. The results for MTT and NR tests are presented in Figure 6.

As can be observed, under H_2_O_2_ conditions, the number of viable cells was reduced, and dehydrogenase activity decreased significantly compared to untreated cells. Pretreatment with ObF ameliorated the negative effects of H_2_O_2_ in a concentration-dependent manner. At the highest tested concentration (150 µg/mL), cell viability was restored to control level.

To verify whether the protective effect is associated with the impact on the cellular enzymatic antioxidant system, the influence on superoxide dismutase (SOD), catalase (CAT), and lipid peroxidation (LO) was assessed. LO was investigated based on the measurement of malondialdehyde (MDA), the main product of lipid peroxidation.

**Figure 7:**
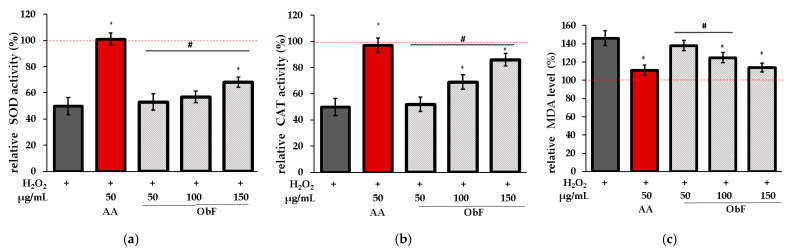
Effect of different concentrations of the polyphenolic fraction from *O. basilicum* (ObF) on antioxidant enzyme activity in H_2_O_2_-treated cells, expressed as a percentage relative to the untreated control. (**a**) relative activity of superoxide dismutase (SOD), (**b**) relative activity of catalase (CAT), (**c**) relative malondialdehyde (MDA) level. The control was 0.5% DMSO in medium. AA—ascorbic acid. * indicates statistically significant difference vs. H_2_O_2_-stimulated cells; # indicates statistically significant difference vs. AA treated cells (*p* < 0.05), the red line was set at 100%.

Figure 7 shows the results obtained for cells pretreated with ObF prior to H_2_O_2_ exposure.

It was found that ObF at 150 µg/mL significantly affected SOD and CAT activity in H_2_O_2_-stimulated normal human colon epithelial cells, while the lower concentration showed no activity for SOD. Furthermore, ObF at concentrations of 100 and 150 µg/mL significantly reduced MDA levels, demonstrating a protective effect against lipid peroxidation. The effectiveness of ObF at 150 µg/mL was comparable to that of ascorbic acid.

## 3. Discussion

*O. basilicum* is a valuable species used worldwide as a dietary additive, not only for its flavor but also for its numerous health benefits and biological potential. This is due to the presence of many phytochemicals, including polyphenolic compounds [24,25,26]. In our paper, biological activity of a polyphenolic-rich fraction isolated by SPE from basil ethanol extract (ObF) was examined.

Analysis of the chemical composition of the fraction showed that it contained primarily different caffeic acid derivatives, with rosmarinic acid and chicoric acid as the predominant constituents. This is consistent with literature reports, which identify these compounds as the main phenolic components of *O. basilicum*’s aerial parts [25,27].

Our study showed that ObF up to 150 µg/mL had no negative impact on human normal colon epithelial cells (CCD 841 CoTr). However, at 200 µg/mL, adverse effects on the cell cycle and apoptosis rate were observed. At this concentration, no cytotoxicity was detected in the NR and MTT assays, which can be explained by the fact that both methods are ineffective at detecting early signs of cell death. The MTT method is based on mitochondrial dehydrogenase activity, which can remain active even as cells are dying, while the NR method assesses membrane integrity. Thus, the detection of cell death with these methods is delayed. In contrast, the cytometric assay identifies early signs of cell death through specific changes in the cell membrane, which occur at the very onset of cell death. As a result, cytometry detects these early stages, leading to an immediate recording of mortality rates.

The cytotoxicity of higher concentrations of basil extracts has also been demonstrated previously. For example, Güez et al. established that the LD_50_ of hydroalcoholic extract in human leukocyte cultures was 35.44 μg/mL [28]. In turn, the safe concentration of crude methanol extract in human peripheral blood mononuclear cells (PBMC) was found to be 30 µg/mL [29]. Furthermore, Faur et al. observed a slight but statistically significant decrease in cell viability at a hydroalcoholic extract concentration of 75 µg/mL in various skin cells, including human keratinocytes, fibroblasts, melanocytes, and mouse epidermis [19]. Considering the concentrations of basil extracts used by other researchers compared to our study, it seems that the polyphenolic fraction, in which the matrix of lipophilic components has been reduced, is less toxic; however, the sensitivity of the cells may also have significance.

Since several reports suggest the existence of basil’s anticancer activity against various cell lines, including human melanoma, human breast cancer, cervical cancer and leukemic cells [19,20,21,22,30], our study examined the impact of ObF on adenocarcinoma (HT-29) cells. Our investigation revealed only a minor effect of the fraction on cell viability. ObF slightly suppressed cancer cell proliferation, and at 150 µg/mL, cell viability assessed by MTT and NR assays decreased to approximately 80% compared to the control. Simultaneously, at this concentration, no reduction in the viability of normal CCD 841 CoTr cells was observed. The relatively low cytotoxicity of the polyphenolic fraction may be due to the lack of essential oil components and triterpenes, such as ursolic acid and lupeol, which are recognized as strong anticancer agents [31,32,33]. It is worth mentioning that significant cytotoxicity reported in the literature was observed for n-hexane:ethyl acetate or crude methanol/ethanol extracts which contain many lipophilic constituents [19,20,21,22].

Since plant phenolics are known for their strong antioxidant potential, high antioxidant effects were expected from ObF. The ability to neutralize free radicals prevents excessive ROS accumulation in cells and alleviates the negative effects of oxidative stress, including lipid and protein oxidation and DNA damage. Therefore, it slows the aging process, protects against chronic diseases and inflammation, supports the immune system, and prevents cancer development [34]. Antioxidants can act directly by reacting with radical species and may support the endogenous antioxidant enzyme system responsible for maintaining redox balance. Our study showed that ObF acts in a dual manner. It indicated ROS scavenging effects in DPPH assay and demonstrated ferric reducing power (FRAP), as well as preventing the depletion of superoxide dismutase (SOD) and catalase (CAT) under oxidative stress conditions. It also reduced the level of malondialdehyde (MDA), which is an indicator of lipid peroxidation. This findings is in accordance with literature data on the antioxidant activity of basil leaf extract demonstrated in various chemical assays, including DPPH, ABTS [19,27,35,36,37], FRAP, [27,35], and oxygen radical absorbance capacity (ORAC) tests [27]. The impact of basil extract on the antioxidant enzyme system was also noted by Güez et al., who found that it affected CAT and MDA levels in leukocytes treated with H_2_O_2_, but did not observe any influence on SOD [28].

It is believed that the anti-inflammatory action of basil is primarily associated with its essential oil components, and the effect of volatile compounds, including estragole, methyl cinnamate, methyl eugenol, α-bergamotene, α-cadinol, and linoleic acid from *O. basilicum*, is well documented [38,39,40,41]. Less is known about the activity of the extract. It has been found that the leaf extract decreases NO levels, an important pro-inflammatory mediator, in LPS-stimulated macrophage cells [17,42], human leukocytes [28], and peripheral blood mononuclear cells [29]. It also elevates the production of the anti-inflammatory cytokine IL-10, which contributes to the regulation of the inflammatory process and helps maintain balance in the inflammatory response [28,42]. Reports on other cytokines are inconsistent. For example, Güez et al. found that basil had no effect on the production of pro-inflammatory cytokines IL-6 and TNF-α [28]. Mueller et al. reported that IL-6 was significantly reduced by *O. basilicum* extract in LPS-stimulated macrophages [42]. In turn, Selvakkumar et al. observed that it downregulated IL-2, TNF-α, and IL-1β in LPS-induced human PBMCs [29]. Furthermore, it has been reported that basil extract may reduce the expression of cyclooxygenase-2, which stimulates prostaglandin production, contributing to the anti-inflammatory action of *O. basilicum* [28,42]. Our results partially confirm previous reports, with a significant decrease in IL-1β, similar to Selvakkumar et al. [29], and an increase in IL-10, like in study of Mueller et al. and Güez et al. [28,42], being observed. However, these effects were noted only in adenocarcinoma cells and no impact on interleukins was demonstrated in normal cells. This observation is important in the context of the anticancer properties of basil, as neoplastic changes lead to the release of chemokines and cytokines, and inflammatory processes disrupt the tissue microenvironment, which promotes cancer development. Despite our study not showing direct, significant cytotoxic effects of ObF on HT-29 cells, the downregulation of IL-1β, a pro-inflammatory cytokine that stimulates neoplastic growth, may support this type of activity.

The activity of ObF is probably linked to the presence of rosmarinic acid (ester of caffeic and 3,4-dihydroxyphenyl lactic acid), and chicoric acid (ester of caffeic and tartaric acids), which are known for their biological potential [43,44,45]. Many scientific papers have described the strong anti-inflammatory effects of rosmarinic acid, among others, through the reduction in cyclooxygenase-2 (COX-2), inducible nitric oxide synthase (iNOS), and pro-inflammatory cytokines IL-6, IL-1, and IL-22. Furthermore, it has the ability to protect against lipopolysaccharide (LPS)-induced inflammation [46,47,48]. Rosmarinic acid is also effective in protecting against oxidative stress and reducing ROS levels. Additionally, it suppresses lipid peroxidation and may enhance the endogenous antioxidant defense system via the expression of catalase, heme oxygenase, and superoxide dismutase [48,49,50,51].

Chicoric acid is also known for its antioxidant potential. It has a high ROS scavenging capacity and protects the cells from free radical-induced cytotoxicity. It has been found that it affects cellular antioxidant systems, including glutathione, glutathione peroxidase, superoxide dismutase, heme oxygenase, and NAD(P)H dehydrogenase in various cell cultures. It also showed anti-inflammatory activity by downregulating iNOS, COX-2, prostaglandin E2, tumor necrosis factor alpha (TNF-α), and interleukins: IL-1β, IL-12, and IL-18. Furthermore, it ameliorated LPS-stimulated inflammation in cell culture and animal models [52,53,54,55,56].

It is important to note that while cell-based models are useful for examining biological effects, they lack the complexity of in vivo systems and, therefore, do not fully reflect the dynamic processes in living organisms, such as metabolism, absorption, and tissue distribution. Additionally, the bioavailability of polyphenols should be considered when assessing their effectiveness in the body, as it can vary depending on factors such as chemical structure, the food matrix, and an individual’s gut microbiota. Consequently, the health benefits observed in vitro may not always translate directly to in vivo outcomes. Therefore, further studies on the activity of basil could include analyzing extracts obtained through simulated digestion, as this would more accurately reflect the conditions under which polyphenols are released and absorbed in the human body.

## 4. Materials and Methods

### 4.1. Plant Material and Extract Preparation

*O. basilicum* leaves were obtained in August 2023 from greenhouses at UMCS in Lublin (voucher specimen: 75_2022). The leaves were washed, air-dried, and subsequently freeze-dried (Christ Alpha 2–4 LDplus dryer, Martin Christ Gefriertrocknungsanlagen, GmbH, Osterode am Harz, Germany). The freeze-dried material was pulverized, weighed and extracted with an accelerated solvent system (ASE) using 80% ethanol in water. The extract was evaporated until dry, and the polyphenolic fraction (ObF) was isolated using solid phase extraction (SPE) with LiChrolut^®^ RP-18 SPE tubes (Merck, Darmstadt, Germany).

### 4.2. Chromatographic Condition

Solvents and analytical standards were from Merck (Sigma-Aldrich Co., St. Louis, MO, USA). Deionized water was obtained using Ultrapure Milipore DirectQ 3UV-R (Merck KGaA, Darmstadt, Germany). Ultra-high performance liquid chromatograph (UPLC) Infinity Series II with a photodiode (DAD) and mass spectrometry (MS) detector (Agilent Technologies, Santa Clara, CA, USA) was used for extract profiling. Chromatography was carried out using a Titan column (10 cm length, 2.1 mm i.d., 1.9 m particle size) (Supelco, Sigma-Aldrich, Burlington, MA, USA). The mobile phase is composed of water (A) and acetonitrile (B), both acidified with 0.1% at flow rate of 0.2 mL/min. The gradient program was as follows: 0–8 min from 98% A to 93% A; 8–15 min from 93% A to 88% A; 15–29 min from 88% A to 85% A; 29–40 min from 85% A to 80% A; and 40–60 min from 80% A to 65% A. Working parameters for DAD and MS were as described in our previous paper [57].

### 4.3. Biological Activity Assays

Spectrometric measurements were carried out using a microplate reader (BioTek Instruments, Winooski, VT, USA). FACS Calibur (BD Pharmingen^TM^, Franklin Lakes, NJ, USA) with CellQuest Pro Version 6.0 software for the Macintosh operating system (BD Pharmingen^TM^) was used for flow cytometric assay. The experimental procedures were performed as previously described [58,59].

#### 4.3.1. Cell Cultures

Human colorectal adenocarcinoma (HT29) cells (ATCC^®^ No. HTB-38™) were cultured in RPMI 1640 medium with 10% fetal calf serum (FCS) (Gibco^TM^, Paisley, UK) and antibiotics (100 U/mL penicillin, 100 g/mL streptomycin, and 0.25 g/mL amphotericin B) (Gibco^TM^, Paisley, UK). Normal colon epithelial (CCD 841 CoTr, SV40 transformed) cells (ATCC^®^ No. CRL-1807™, Manassas, VA, USA) were cultured in RPMI 1640 + DMEM (1:1) medium (Sigma-Aldrich) with 10% FCS and antibiotics. The process was carried out at 37 °C in a humidified atmosphere with 5% CO_2_. The cells were grown for 24 h in 96-well multiplates in 100 µL of culture medium supplemented with different concentrations of extract (25–200 µg/mL). To establish the effects against H_2_O_2_-induced stress, the cells pretreated with the extract were then stimulated with H_2_O_2_ (250 μM) for 30 min [60].

#### 4.3.2. Cell Viability

MTT assay: after 24 h, the medium was discarded and replaced with fresh medium containing MTT solution at concentration of 5 mg/mL (25 µL/well). After 3 h of incubation, the formazan crystals were solubilized overnight using 10% sodium dodecyl sulfate in 0.01 M HCl. Absorbance was measured at 570 nm.

Neutral Red (NR) uptake assay: after 24 h, the medium was discarded, and 0.4% NR solution in medium was added to each well. After 3 h of incubation, the dye-containing medium was removed, and the cells were fixed with 1% CaCl_2_ in 4% paraformaldehyde (200 μL). Afterwards, the incorporated dye was solubilized using 1% acetic acid in a 50% ethanol solution (100 μL). The plates were gently shaken for 20 min. Absorbance was measured at 540 nm.

#### 4.3.3. Cytometric Analysis of the Cell Cycle

After incubation, the floating and adherent cells were harvested, centrifuged (3000 rpm/5 min), rinsed in PBS w/o Ca^2+^ and Mg^2+^ ions, once again centrifuged, and fixed in 70% ethanol. The samples were stored for 1 week in −20 °C. After this time, the samples were subjected to PI staining (PI/RNase Staining Buffer, BD Pharmingen^TM^, BD Biosciences, San Jose, CA, USA). The results were calculated as a percentage of cells in the respective cell cycle phases (sub-G1, G0/G1, S and G2). In total, 10,000 events were measured per sample.

#### 4.3.4. Flow Cytometry

HT29 or CCD 841 CoTr cells were seeded into 6-well plates at a density of 1 × 10^5^ cells/well. After cell adhesion (the next day), the growth medium was replaced with a fresh one containing extracts. After 24 h incubation, the floating and adherent cells were harvested, washed with PBS without Ca^2+^ and Mg^2+^ ions, and suspended in 1 × binding buffer. The cells were stained with 5 mM of FITC-Annexin V and 5 mM of PI using an Annexin V-fluorescein isothiocyanate (FITC)/propidium iodide (PI) apoptosis kit (BD Biosciences, BD Pharmingen™, San Jose, CA, USA). After 15 min of incubation in the dark at room temperature, the cells were immediately analyzed.

#### 4.3.5. May–Grünwald–Giemsa (MGG) Staining

After 24 h of incubation, the medium was discarded, the cells were rinsed with culture medium and stained with May–Grünwald (MG) stain for 5 min, followed by staining for another 5 min in MG diluted in an equal quantity of water. The MG was removed and Giemsa reagent (diluted 1:20 in water) was added to the cells, which were next incubated at room temperature for 15 min. Thereafter, the cells were rinsed, dried, and subjected to microscopic observations (Olympus, BX51; Olympus, Tokyo, Japan).

#### 4.3.6. ELISA Assay

The levels of human interleukins including IL-1β, IL-6, IL-10 (Elabscience, Houston, TX, USA), and the MDA-level activity of SOD and CAT (Abcam, Berlin, Germany) were measured immunoenzymatically (ELISA) using commercially available kits according to the manufacturer’s instructions.

#### 4.3.7. Antioxidant Assay

Free radical scavenging activity (DPPH) and ferric reducing antioxidant power (FRAP) assays were carried out using procedures described previously [58]. Briefly, 100 µL of DPPH at a concentration of 0.2 mg/mL was added to 100 µL of the fraction at various concentrations and to increasing concentrations of Trolox (Sigma) used as a reference. The samples were incubated for 20 min at room temperature, and the absorbance was measured at 515 nm. For the FRAP test, the fraction at various concentrations was mixed with an equal volume of 0.2 M sodium phosphate buffer (pH 6.6), and 1% potassium ferricyanide was incubated for 30 min at 37 °C. Next, 10% trichloroacetic acid (*w*/*v*) was added and the solution was centrifuged. A total of 1 mL of the upper layer was mixed with an equal volume of water and 0.1% ferric chloride and the absorbance was measured at 700 nm.

### 4.4. Statistical Analysis

Results are presented as means ± SD from three experiments. Data were analyzed using one-way ANOVA with Dunnett’s post hoc test. Differences of *p* < 0.05 were considered significant.

## 5. Conclusions

Our investigation shows that *O. basilicum* is a rich source of caffeic acid derivatives, in particular rosmarinic acid and chicoric acid. Furthermore, the polyphenolic fraction *of O. basilicum* exhibits strong free radical scavenging activity and reducing antioxidant power in DPPH and FRAP tests, protects cells against H_2_O_2_-induced cytotoxicity, and maintains cellular redox balance by preventing the depletion of antioxidant enzymes, including SOD and CAT. It also prevents lipid peroxidation, as evidenced by a decrease in MDA levels. Despite showing no cytotoxicity against human colorectal adenocarcinoma (HT-29), basil polyphenolic extract decreases the pro-inflammatory cytokine IL-1β in a neoplastic environment, indicating its potential role in cancer prevention. Therefore, it can be concluded that the consumption of basil may contribute to health benefits, including alleviating oxidative stress and supporting the immune system.

## Figures and Tables

**Table 1:** Mass data and the results of quantification of the main components in the isolated fraction from *O. basilicum* expressed as mg/g ± standard deviation of dried extract.

R_t_ (min.)	Mass Data (*m*/*z*-H)	Fragment (*m*/*z*-H)	Formula	Component	Content (mg/g)
7.64	311.0405	(135, 179)	C_13_H_12_O_9_	Caftaric acid (str)	2.82 ± 0.21
12.76	341.08655	(179, 135)	C_15_H_18_O_9_	Caffeic acid hexoside	2.71 ± 0.17
13.63	179.03502	(135)	C_9_H_8_O_4_	Caffeic acid (str)	4.58 ± 0.29
16.79	295.04533	(135, 179)	C_13_H_12_O_8_	Caffeoylmalic acid ^1^	2.98 ± 0.20
20.93	473.07402	(135, 149, 161, 179, 293, 311)	C_22_H_18_O_12_	Chicoric acid (str)	15.23 ± 1.01
21.73	473.07389	(135, 149, 161, 179, 293, 311)	C_22_H_18_O_12_	Chicoric acid isomer	5.16 ± 0.31
28.64	487.08856	(135, 161, 179, 193)	C_23_H_20_O_12_	Caffeoyl feruloyl tartaric acid ^2^	3.05 ± 0.19
31.28	359.07583	(135, 161, 179, 197)	C_18_H_16_O_8_	Rosmarinic acid isomer	2.01 ± 0.18
33.86	359.07669	(135, 161, 179, 197)	C_18_H_16_O_8_	Rosmarinic acid (str)	27.23 ± 1.45

str—identification confirmed using standard; ^1^—quantification based on caffeic acid; ^2^—quantification based on rosmarinic acid.

**Table 2:** Cytokine concentration (pg/mL) after 24 h incubation of human normal colon epithelial cells (841CoTr) and human colorectal adenocarcinoma cells (HT29) with polyphenolic fraction from *O. basilicum* (ObF) at 150 µg/mL concentration.

Cytokine	841 CoTr		HT29	
	Control	ObF	Control	ObF
IL-1β	1863.8 ± 167.2	2113.0 ± 186.5	1835.6 ± 109.1	1318.9 ± 65.3 *
IL-6	1707.5 ± 31.8	1797.5 ± 89.4	1677.5 ± 148.5	1700.0 ± 128.5
IL-10	1353.8 ± 111.4	1381.0 ± 82.5	1140.6 ± 117.0	1302.2 ± 98.3 *

* indicates a statistically significant difference at *p* < 0.05 using one-way ANOVA followed by Dunnett’s post hoc test.

**Table 3:** DPPH and FRAP results (*n* = 3) ±SD obtained for *O. basilicum* polyphenolic fraction.

Concentration of the Fraction (µg/mL)	DPPH (Equivalent of Trolox Concentration) *	FRAP (Equivalent of Ascorbic Acid Concentration) *
50	10.45 ± 0.46	5.41 ± 0.23
100	22.11 ± 0.62	32.22 ± 0.46
150	34.31 ± 0.98	47.54 ± 1.07
200	52.21 ± 1.52	66.19 ± 1.76

* the reducing/antioxidant power of the extract at a given concentration is equivalent at a given concentration of ascorbic acid/Trolox.

## Data Availability

The data presented in this study are available on request from the corresponding author.

## Supplementary Materials

The following supporting information can be downloaded at https://www.mdpi.com/article/10.3390/molecules29215043/s1: Table S1. Mass data of the compounds found in the extracts from *Ocimum basilicum*; Figure S1: Overlapped base peak chromatograms (BPC) of ethanol–water extract of Ocimum basilicum (green line) and the polyphenolic fraction isolated from ethanol–water extract (red line); Figure S2: Extracted ion chromatogram (EIC) in the range of 179.034–179.036 *m*/*z* of the polyphenolic fraction isolated from ethanol–water extract of *Ocimum basilicum* and UV-Vis spectra of the main components.
