# Supplementary material for: Ultra-Performance Liquid Chromatography and Mass Spectrometry Characterization, and Antioxidant, Protective, and Anti-Inflammatory Activity, of the Polyphenolic Fraction from Ocimum basilicum
Source: Molecules. 2024 Oct 25;29(21):5043. doi: 10.3390/molecules29215043 (PMC11547609; doi:10.3390/molecules29215043)
Supplement: Supplementary file 1 [file molecules-29-05043-s001.zip › molecules-3272511-supplementary.pdf]

molecules

MDPI

Article

UPLC-MS Characterization and Antioxidant, Protective, and Anti-Inflammatory Activity of the Polyphenolic Fraction from *Ocimum basilicum*

Magdalena Wójciak [1]*, Roman Paduch [2,3], Piotr Drozdowski [4], Magdalena Żuk [1], Weronika Wójciak [1], Katarzyna Tyszczuk-Rotko [5], Marcin Feldo [6] and Ireneusz Sowa [1]

[1] Department of Analytical Chemistry, Medical University of Lublin, Chodźki 4a, 20-093 Lublin, Poland;
[2] Department of Virology and Immunology, Institute of Biological Sciences, Faculty of Biology and Biotechnology, Maria Curie-Skłodowska University, 19 Akademicka Street, 20-033 Lublin, Poland;
[3] Department of General and Pediatric Ophthalmology, Medical University of Lublin, Chmielna 1, 20-079 Lublin, Poland
[4] Department of Plastic Surgery, Specialist Medical Centre, 57-320 Polanica-Zdrój, Poland (P.D.)
[5] Faculty of Chemistry, Institute of Chemical Sciences, Maria Curie-Skłodowska University in Lublin, 20-031 Lublin, Poland
[6] Department of Vascular Surgery, Medical University of Lublin, Staszica 11 St., 20-081 Lublin, Poland;

Table S1. Mass data of the compounds found in the extracts from *Ocimum basilicum*.

R_T (min.)	Mass Data (m/z-H)	Fragment (m/z-H)	Formula	Δ ppm	Component
1.56	191.05632		$C_7H_{12}O_6$	1.08	Quinic acid (str)
1.66	133.01471		$C_4H_6O_5$	3.46	Malic acid (str)
2.01	191.01983		$C_6H_8O_7$	0.54	Citric acid (str)
7.64	311.0405	(135,179)	$C_{13}H_{12}O_9$	-1.14	Caftaric acid (str)
7.84	203.08204		$C_{11}H_{12}N_2O_2$	-2.75	Tryptophan (str)
12.09	417.09061	(221)	C13H22O15	4.82	unidentified
12.44	401.18237	(221)	C19H30O9	1.65	unidentified
12.76	341.08655	(179,135)	C15H18O9	-3.67	Caffeic acid hexoside
13.63	179.03502	(135)	$C_9H_8O_4$	0.21	Caffeic acid (str)
16.23	387.16687	(207)	C18H28O9	2.10	Tuberonic acid glucoside
16.79	295.04533	(135, 179)	$C_{13}H_{12}O_8$	-2.06	Caffeoylmalic acid
18.39	357.0618	(269, 313)	C17H14O6	0.58	unidentified
20.93	473.07402	(149,179,293,311)	C22H18O12	3.10	Chicoric acid (str)
21.73	473.07389	(149,179,293,311)	C22H18O12	2.83	Chicoric acid isomer
28.64	487.08856	(135,161,179,193)	C23H20O12	0.74	Caffeoyl feruloyl tartaric acid
31.28	359.07583	(135,161, 179,197)	C18H16O8	-3.92	Rosmarinic acid isomer
33.16	719.16028	(519,555)	C36H32O16	-2.05	Rashomonic acid
33.86	359.07669	(135,161, 179,197)	C18H16O8	-1.53	Rosmarinic acid (str)
36.73	717.14802		C36H30O16	2.66	Salvianolic acid (str)

str - identification was confirmed using standard

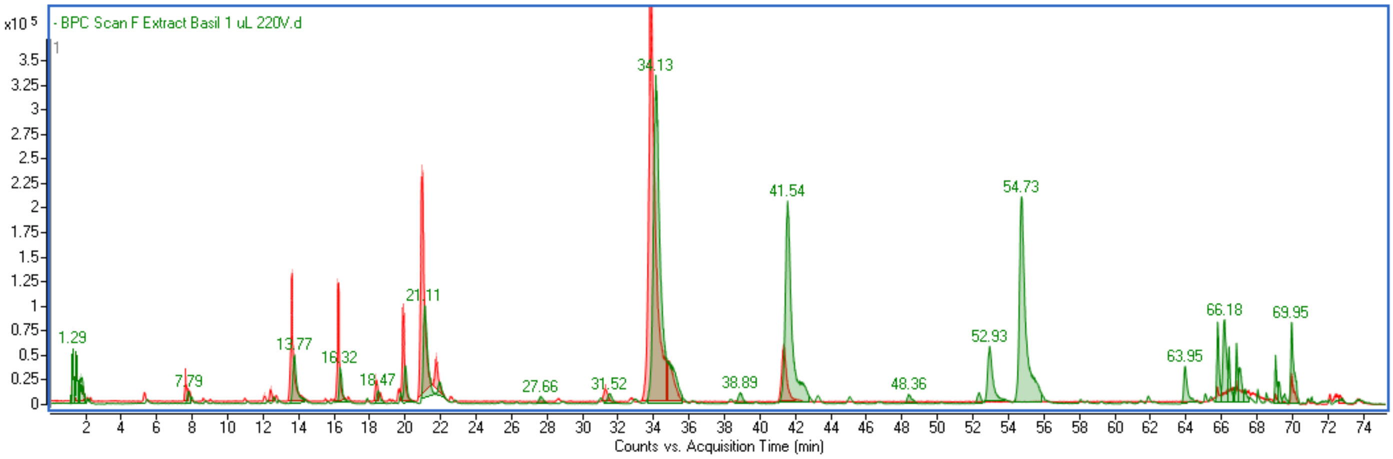

Figure S1. Overlapped base peak chromatograms (BPC) of ethanol-water extract of *Ocimum basilicum* (green line) and the polyphenolic fraction isolated from ethanol-water extract (red line).

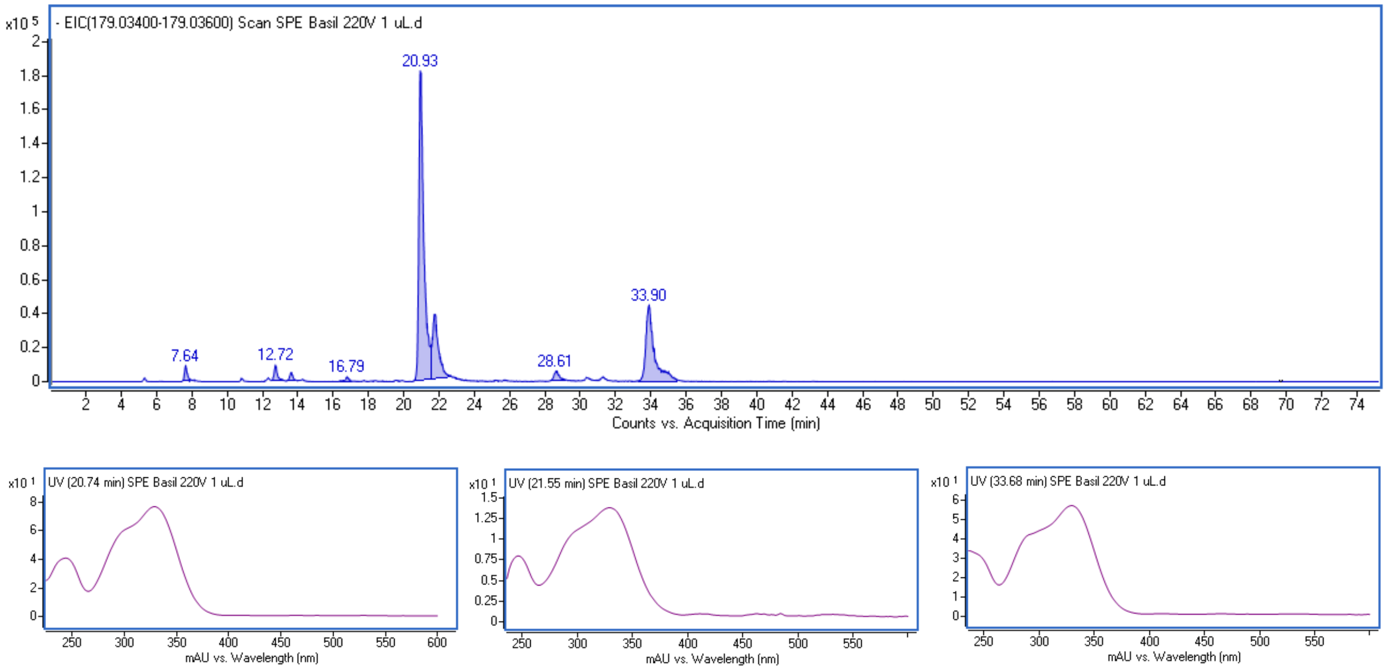

Figure S2. Extracted ion chromatogram (EIC) in the range of 179.034-179.036 m/z of the polyphenolic fraction isolated from ethanol-water extract of *Ocimum basilicum* and UV-Vis spectra of the main components
